# WHO target product profiles: four diagnostic tests needed in the effort to eliminate African trypanosomiasis

**DOI:** 10.2471/BLT.23.290106

**Published:** 2023-06-26

**Authors:** Gerardo Priotto, Jose R Franco, Veerle Lejon, Philippe Büscher, Enock Matovu, Joseph Ndung'u, Sylvain Biéler, Dieudonné Mumba, Nick Van Reet, Paul Verlé, Vincent Jamonneau, Pere P Simarro, Augustin Kadima Ebeja, Dieudonné Sankara, Daniel Argaw Dagne

**Affiliations:** a Department of Control of Neglected Tropical Diseases, World Health Organization, avenue Appia 20, 1211 Geneva 27, Switzerland.; b Institut de Recherche pour le Développement, CIRAD, University of Montpellier, France.; c Institute of Tropical Medicine, Antwerp, Belgium.; d College of Veterinary Medicine, Animal Resources and Biosecurity, Makerere University, Kampala, Uganda.; e Foundation for Innovative New Diagnostics-Kenya, Nairobi, Kenya.; f Neglected Tropical Diseases Programme, Foundation for Innovative New Diagnostics, Geneva, Switzerland.; g Department of Parasitology, Institut National de Recherche Biomédicale, Kinshasa, Democratic Republic of the Congo.; h Communicable Disease Unit, World Health Organization Regional Office for Africa, Brazzaville, Congo.

Human African trypanosomiasis, also known as sleeping sickness, is a life-threatening parasitic infection transmitted by the tsetse fly in sub-Saharan Africa. Although it caused devastating epidemics during the 20th century, its incidence has now fallen to historically low levels^1^ thanks to sustained and coordinated efforts over the past two decades.

Two trypanosome subspecies cause the disease: *Trypanosoma brucei gambiense* and *T. b. rhodesiense*. Between 2011 and 2020, *T. b. gambiense* accounted for 95% (32 275 out of 34 096 reported cases) of the total caseload of human African trypanosomiasis.^1^ *T. b. gambiense* is present in western and central Africa, with humans as the main reservoir. *T. b. rhodesiense* manifests in frequent epidemic seasonal outbreaks in eastern and southern Africa and is occasionally transmitted to humans from its wild and domestic animal reservoirs. *T. b. rhodesiense* causes an acute illness with clinical onset generally a few weeks after infection that develops rapidly, often provoking multiorgan failure and invading the central nervous system.

The distinct epidemiology of gambiense trypanosomiasis versus rhodesiense trypanosomiasis leads to different control strategies. Control of gambiense trypanosomiasis is based on screening at-risk populations for case finding and subsequent treatment to decrease the reservoir, complemented by targeted vector control. This strategy has led to a 98% incidence reduction (from 26 574 to 663 cases) between 2000 and 2020.^1^ In contrast, rhodesiense trypanosomiasis is harder to eliminate because of its animal reservoir, and control methods rely mostly on vector and animal health interventions – with variable success. Nevertheless, early diagnosis and treatment of cases of this subtype can considerably reduce the impact on human health.

Several tools are available for the screening and diagnosis of gambiense trypanosomiasis, but tools for rhodesiense trypanosomiasis are either missing or, if they exist, are losing ground in the evolving context of health services in rural Africa.

Human African trypanosomiasis diagnosis relies on laboratory techniques because clinical signs and symptoms are unspecific. Serodiagnostic field tests exist only for *T. b. gambiense*. As these tests detect antibodies, they are not confirmatory of infection, and in the current low disease prevalence, their positive predictive value is low. Field-applicable tools include the card agglutination test used mainly in active screening by mobile teams, and rapid diagnostic tests more suitable for point-of-care individual testing. Confirmation of a *T. b. gambiense* infection requires microscopic examination of body fluids (blood, lymph and cerebrospinal fluid), which requires specific training on microscopy techniques. The best- performing parasite detection methods are laborious and complex, and reach 85%–95% diagnostic sensitivity when performed by skilled personnel. Because in most methods, human African trypanosomes are identified visually by their characteristic movement, microscopic examination must be done within less than 1 hour after sampling.

While no simple serological tests for rhodesiense trypanosomiasis exist, the infection is easier to detect by microscopy due to its higher parasitaemia. However, the introduction of rapid diagnostic tests for malaria has resulted in decreased capacity for microscopy examinations in peripheral health facilities. Consequently, the accidental diagnosis of rhodesiense trypanosomiasis, which was common when microscopy was routinely done for malaria, decreased. In many endemic areas, rhodesiense trypanosomiasis diagnosis is often missed or made too late, resulting in increased mortality.

Both forms of human African trypanosomiasis have been targeted for elimination as a public health problem,^2^ defined as a five-year mean of less than  one case per 10 000 inhabitants in all endemic districts in a country. Several countries have eliminated the disease as a public health problem, as validated by the World Health Organization (WHO). The next target is the elimination of transmission of *T. b. gambiense*, defined as zero autochthonous cases for at least five consecutive years.^3^ Countries reaching either of these goals still need to maintain dedicated surveillance because of the persisting risk of re-emergence or reintroduction.

Unsurprisingly, the progress in human African trypanosomiasis elimination is leading to an unintended gradual loss of specialized personnel. This shortage calls for simple diagnostic methods that can be performed by non-specialized personnel, because currently available diagnostic tools are complex and resource-intensive.

In this rapidly changing context, the WHO Department of Control of Neglected Tropical Diseases launched a process to identify and prioritize diagnostic needs. A WHO Neglected Tropical Diseases Diagnostics Technical Advisory Group was formed, with different subgroups working on specific neglected tropical diseases. The subgroup of independent experts working on human African trypanosomiasis comprises leading international scientists and specialists, including from countries where the disease is endemic.. The work of the advisory group led to the development of target product profiles for trypanosomiasis diagnostic tests.

In order of priority, four target product profiles have been developed.

First, a profile for a test for rhodesiense trypanosomiasis diagnosis usable in peripheral health facilities.^4^ In addition to allowing faster prescription of a treatment, a simple test would also help in capturing more information on the occurrence of *T. b. rhodesiense* transmission, hence recovering the loss of surveillance capacity and possibly strengthening this capacity beyond the previous levels. To do so, a *T. b. rhodesiense* test should be practicable at the locations where people seek malaria diagnosis. Ideally, it should be an antigen detection test in a simple format, but it could also be a simple molecular test detecting genetic material, or a microscopy-free method detecting the presence of human African trypanosomes. The test should provide immediate results granting therapeutic decisions by confirming the infection, but, if not possible, it could be a screening test to be followed by confirmatory microscopic examination – considering that parasitaemia is usually high. In the current context, confirmation is mandatory, but in a future with a safer medicine, an appropriate screening test could grant immediate treatment.

Second, a gambiense trypanosomiasis test to identify highly suspected individuals to receive treatment is needed.^5^ Repeated rounds of screening for *T. b. gambiense* followed by treatment of cases detected can reduce the prevalence to low levels, and this approach has been the cornerstone strategy of gambiense trypanosomiasis control and elimination. However, among the seropositive but microscopically unconfirmed individuals, a variable proportion harbours the parasite and could perpetuate the reservoir. Recommending treatment on the basis of suspicion alone has not been possible so far, because current treatments are logistically challenging and not sufficiently safe. Current treatments have considerable toxicity and increase the risk of other infections; therefore, disease diagnosis needs to be confirmed to treat. The expected advent of a safer and easy-to-use treatment, currently in phase 3, would favourably tip the benefit–risk balance. A simple diagnostic tool to identify individuals eligible for treatment would be the ideal complement for this powerful elimination approach. The envisioned tool could be any method of high sensitivity but simple enough to be applicable at the point-of care, including in mobile laboratories at village level in zero infrastructure conditions, requiring minimal specialized training. The tool should identify individuals with a high degree of suspicion of infection (independently of symptoms) that can be considered sufficient to justify treatment with a medicine that has a good safety profile. Ideally, a therapeutic decision could be reached with one test. A tandem of two simple sequential tests would also be acceptable.

Third, a gambiense trypanosomiasis individual test to assess infection in low prevalence settings.^6^ In the context of human African trypanosomiasis elimination, this tool would solve two issues. First, confirming *T. b. gambiense* infection in field-level settings where microscopy has become absent or unreliable due to the scarcity of cases, leading to progressive lack of experience. Second, assessing *T. b. gambiense* infection a posteriori among individuals who have received presumptive treatment on the spot. Implemented in national or subnational reference laboratories, the test would be performed on samples transferred from the field settings. Sampling should ideally be blood from finger-prick, but acceptable as serum, plasma or blood in a carrier ensuring stability for 4 weeks at 40 °C and 12 months at 4 °C. Importantly, within a potential strategy of widened treatment with the foreseen oral, single-dose safe medicine, this test would be essential to monitor the epidemiological situation and to adjust the programme accordingly. Moreover, this tool would provide key data to assess the elimination status, towards the elimination targets.

Fourth, a gambiense trypanosomiasis high-throughput test for verification of elimination.^7^ This method should be able to simultaneously analyse numerous samples collected in remote rural areas, and ideally be performed in national or subnational reference laboratories. If applicable also in animals, this method could further help assessing the parasite circulation in an area. Specimen collection should be simple, with no cold chain needed for the transfer to reference laboratories. The incidence of gambiense trypanosomiasis has been strongly declining, and some historically endemic countries and/or foci have not reported cases for years. Therefore, the need is increasing for high-throughput methods for population-level cross-cutting surveillance of *T. b. gambiense* transmission. This tool would allow for testing at-risk populations with comprehensive coverage, and populations thought to have become risk-free where absence of transmission needs verification.

These target product profiles are intended to highlight the diagnostic tools needed for the future strategies to combat human African trypanosomiasis, and to facilitate the decision processes for potential product developers and donors.
